# A Full-Length Transcriptome and Analysis of the NHL-1 Gene Family in *Neocaridina denticulata sinensis*

**DOI:** 10.3390/biology13060366

**Published:** 2024-05-22

**Authors:** Kefan Xing, Huimin Li, Xiongfei Wang, Yuying Sun, Jiquan Zhang

**Affiliations:** 1 School of Life Sciences/Hebei Basic Science Center for Biotic Interaction, Hebei University, Baoding 071002, China; xingkefanaa@163.com (K.X.); 18709865271@163.com (H.L.); 17732104202@163.com (X.W.); 2 Institute of Life Science and Green Development, Hebei University, Baoding 071002, China

**Keywords:** full-length transcriptome, *Neocaridina denticulata sinensis*, NHL-1, phylogenetic analysis

## Abstract

**Simple Summary:**

This study described a full-length transcriptome of *Neocaridina denticulata sinensis* that was generated using third-generation sequencing technology. The resulting transcriptome assembly comprised 5831 transcripts, revealing 90.5% novel isoforms of known genes. A functional annotation showed that 24.8% of the transcripts can be searched across seven key databases. Additionally, 1236 alternative splicing events, 344 transcription factors, and 124 long non-coding RNAs were predicted. Notably, a RING finger protein NHL-1 coding gene was characterized with 15 transcripts, and a phylogenetic analysis underscored its evolutionary connection to NHL-1 in other crustaceans. This full-length transcriptome dataset constitutes a valuable genetic resource in *Neocaridina denticulata sinensis* and will facilitate functional genomic studies and investigations into environmental adaptations in this species.

**Abstract:**

*Neocaridina denticulata sinensis* has emerged as a promising model organism for basic studies in Decapod. However, the current transcriptome information on this species is based on next-generation sequencing technologies, which are limited by a short read length. Therefore, the present study aimed to generate a full-length transcriptome assembly of *N. denticulata sinensis* utilizing the PacBio Sequel Ⅱ platform. The resulting transcriptome assembly comprised 5831 transcripts with an N50 value of 3697 bp. Remarkably, 90.5% of these transcripts represented novel isoforms of known genes. The transcripts were further searched against the NR, SwissProt, KEGG, KOG, GO, NT, and Pfam databases. A total of 24.8% of the transcripts can be annotated across all seven databases. Additionally, 1236 alternative splicing events, 344 transcription factors, and 124 long non-coding RNAs (LncRNAs) were predicted. Based on the alternative splicing annotation results, a RING finger protein NHL-1 gene from *N. denticulata sinensis* (*NdNHL-1*) was identified. There are 15 transcripts in *NdNHL-1*. The longest transcript is 4995 bp in length and encodes a putative protein of 1665 amino acids. A phylogenetic analysis showed its close relationship with NHL-1 from other crustacean species. This report represents the full-length transcriptome of *N. denticulata sinensis* and will facilitate research on functional genomics and environmental adaptation in this species.

## 1. Introduction

*Neocaridina denticulata sinensis* is a common freshwater shrimp across East Asia. The species is highly sought after in the world aquarium trade due to its colorful body pigmentation and important ecological function. Despite not being a major aquaculture species, *N. denticulata sinensis* is a promising laboratory organism for basic study in Decapod due to its small size, short life cycle, and ease of rear [1]. However, previous research mostly focused on reproduction, toxicology, and functional genes [2,3]; therefore, genetic information on this shrimp remains limited. Full-length transcripts are powerful tools for conducting studies in structural and functional genomics. Although second-generation sequencing technology has been used to perform transcriptome studies in this species, the short-read RNA-seq data remains a barrier to the assembly of a full-length transcriptome.

Single-molecule real-time (SMRT) sequencing is a type of third-generation sequencing technology; it provides long, highly accurate sequencing reads that enhance the assembly of complete genomes [4]. SMRT sequencing has been applied in many species [5,6,7]. Full-length transcriptomes have been reported for several crustacean species, including *Penaeus monodon* [8], *Portunus trituberculatus* [9], *Scylla paramamosain* [10], and *Marsupenaeus japonicus* [11]. Nevertheless, the full-length transcriptome of *N. denticulata sinensis*, an important genomic resource, has not yet been explored.

The present study described the first full-length transcriptome assembly derived from multiple tissues of *N. denticulata sinensis* through Pacific Biosciences (PacBio) SMRT sequencing technology. A functional annotation of the full-length transcripts was conducted. Furthermore, the prediction of alternative splicing events, transcription factors, and long non-coding RNAs (LncRNAs) were performed. RING finger protein NHL-1 is involved in protein ubiquitination, which is a highly conserved and fundamental intracellular protein modification process [12]. Therefore, the gene structure and phylogenetic relationship of *N. denticulata sinensis NHL-1* (*NdNHL-1*) were analyzed based on the sequencing results. This study contributes to the expansion of the transcriptome dataset of *N. denticulata sinensis*, thus facilitating the analysis of the gene structure and post-transcriptional regulation.

## 2. Materials and Methods

### 2.1. Shrimp Culture and Samples

Shrimp were collected from Baiyangdian Lake, Baoding, China and reared in indoor recirculating aquaculture systems (25 ± 1 °C) with adequate aeration. To minimize animal suffering and reduce the number of animals used, ethical considerations were thoroughly followed. Cephalothorax and muscle tissues from six individuals were pooled, including one injected with 2 μL of *Pichia pastoris* (9.1 × 10^6^ cfu/mL), one injected with 2 μL of *Bacillus* (1.4 × 10^7^ cfu/mL), one injected with 2 μL of *Escherichia coli* (2.3 × 10^8^ cfu/mL), one exposed to Cu^2+^ (2.5 μM) for 24 h, one starved for 4 d, and one without treatment. These tissues were dissected and immediately frozen in liquid nitrogen and then stored at −80 °C to ensure optimal preservation of RNA integrity prior to extraction.

### 2.2. RNA Extraction, SMRT Library Preparation, and Sequencing

Total RNA was extracted from pooled samples using the TRIzol Reagent (Invitrogen, Carlsbad, CA, USA) following the manufacturer’s instructions. RNA degradation was assessed on 1% agarose gels. RNA purity and concentration were measured using NanoDrop 2000 (Thermo Scientific, Waltham, MA, USA) and Qubit 2.0 Fluorometer (Life Technologies, Carlsbad, CA, USA). RNA integrity was assessed using the RNA Nano 6000 Assay Kit of the Agilent Bioanalyzer 2100 system (Agilent Technologies, Santa Clara, CA, USA). The cDNA was prepared from pooled tissues using the SMARTer PCR cDNA Synthesis Kit (Takara, Osaka, Japan). Size fractionation and selection were performed using the BluePippin Size Selection System (Sage Science, Beverly, MA, USA) protocol as described by Pacific Biosciences (PN 100-092-800-03). The qualified libraries were sequenced on a PacBio Sequel Ⅱ platform.

### 2.3. Data Processing

Sequence data were processed using the SMRTlink 5.0 software (Pacific Biosciences, Menlo Park, CA, USA). A circular consensus sequence (CCS) was generated from subread BAM files. The CCS contained the 5’ primer; the 3’ primer and poly A tail were considered as a full-length transcript sequence. Full-length and non-full-length sequences were then subjected to isoform-level clustering (ICE), followed by final Arrow polishing. The polished consensus sequences were used for a subsequent analysis. Nucleotide errors in consensus sequences were corrected using the Illumina RNA-seq data with the LoRDEC software V0.7 [13]. Consensus reads were aligned to the reference genome using GMAP [14]. The functional annotation of transcripts was based on the following databases: NR, NT, Pfam, KOG, Swiss-Prot, KEGG, and GO. BLAST software V2.7.1 was used for NT database analysis with the e-value set at 1 × 10^−10^. Diamond BLASTX V0.8.36 was used to analyze the NR, KOG, Swiss-Prot, and KEGG databases with the e-value set at 1 × 10^−10^. Hmmscan V3.1b2 was selected for the Pfam database analysis. GO and KEGG enrichment analyses were implemented using the GOseq R package V1.10.0 [15] and KOBAS software V3.0 [16].

### 2.4. Alternative Splicing, Transcription Factor, and Long Non-Coding RNA Analysis

An alternative splicing analysis was performed using SUPPA V2.3 [17] with default parameters. Transcription factors were predicted using the AnimalTFDB 2.0 database [18]. CNCI V2 (Coding-Non-Coding Index) [19], Pfam-scan V1.6 [20], and PLEK SVM classifier V1.2 [21] were used to predict the coding potential of transcripts with default parameters. A CPC (Coding Potential Calculator) V0.9 [22] was also used to search the sequences with the NCBI eukaryote protein database to identify the coding and non-coding transcripts with the e-value set as 1 × 10^−10^.

### 2.5. Gene Analysis

The sequence of *NdNHL-1* was obtained according to the AS annotation results. The molecular weight and isoelectric point of *NdNHL-1* was calculated using perl scripts. The gene structures of 15 *NdNHL-1* transcripts were drawn using the R package GGGENES v0.5.0 [23]. Specifically, *NdNHL-1* was aligned with MAFFT v7.505 using the ‘--auto’ strategy and normal alignment mode [24]. Maximum likelihood (ML) phylogenies were inferred using IQ-TREE v2.2.0 under the JTT + G4 model for 5000 ultrafast bootstraps, as well as the Shimodaira–Hasegawa-like approximate likelihood ratio test [25].

## 3. Results

### 3.1. Overview of Full-Length Transcript Sequencing Data

The Iso-seq method for *N. denticulata sinensis* was performed using the PacBio Sequel II platform. The resulting full-length transcriptome can be assessed by the size of the sequencing data and characteristic values of the reads and transcripts. A total of 65.65 Gb of polymerase reads was obtained. After removing adapters and reads shorter than 50 bp, 63.96 Gb of subreads was left, with an average length of 2757 bp and an N50 value of 3237 bp. A total of 193,468 CCS were generated, including 36,653 non-full-length sequences and 156,815 full-length sequences. A total of 154,402 (88.84%) full-length nonchimeric (FLNC) reads were obtained. The hierarchical n*log(n) algorithm and Arrow software (https://github.com/PacificBiosciences/pb-assembly, accessed on 15 April 2024) were used to cluster and correct the FLNC reads to generate a consensus sequence for a subsequent analysis; 13,082 consensus sequences were obtained, with the length ranging from 83 bp to 9788 bp (Table 1). Subsequently, the polished consensus sequences were aligned to the *N. denticulata sinensis* reference genome. A total of 12,472 (95.34%) reads can be mapped, and the numbers of multiple mapped and unique mapped reads were 72 (0.55%) and 12,400 (94.79%), respectively. Additionally, 610 (4.66%) reads cannot be mapped to the reference genome; these reads may come from parasites or symbiont on the shrimp. The unmapped sequences were further searched against seven databases, and 553 (91%) of them can be annotated in at least one database.

### 3.2. Classification and Functional Annotation of Transcripts

**Figure 1:**
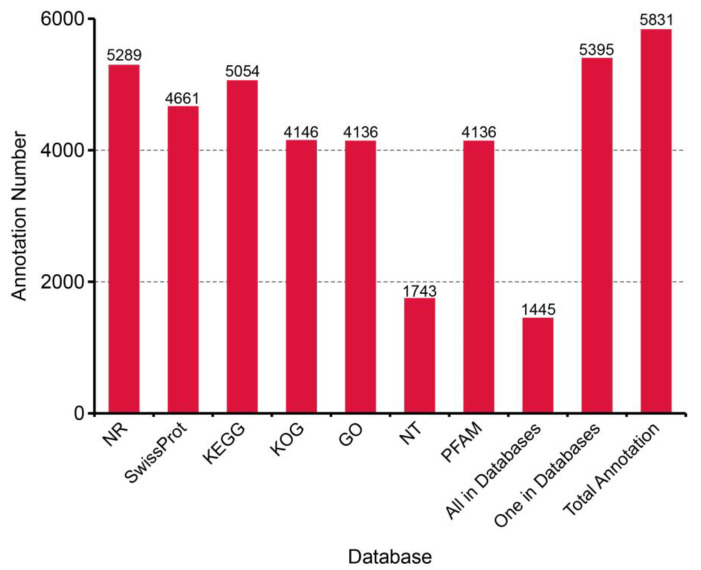
Transcript annotation of *N. denticulata sinensis* in seven databases (databases: NR, SwissProt, KEGG, KOG, GO, NT, and Pfam; “All in databases” represents number of transcripts successfully annotated in seven databases; “One in databases” represents number of transcripts successfully annotated in at least one database; and “Total Annotation” represents number of all transcripts).

A total of 5277 (90.5%) sequences were identified as novel isoforms of known genes, and 486 (8.3%) sequences were classified as isoforms of novel genes. The functional annotation results of transcripts are shown in Figure 1. In general, 1445 (24.8%) transcripts were annotated in all databases. In total, 5289 (90.7%), 4661 (80%), 5054 (86.7%), 4146 (71.1%), 4136 (70.9%), 1743 (29.9%), and 4136 (70.9%) transcripts matched the NR, SwissProt, KEGG, KOG, GO, NT, and Pfam databases. According to the NR annotation, the top three most matched species were *Hyalella azteca* (2224, 42.05%), *Zootermopsis nevadensis* (359, 6.79%), and *Limulus polyphemus* (164, 3.1%) (Table 2).

**Figure 2:**
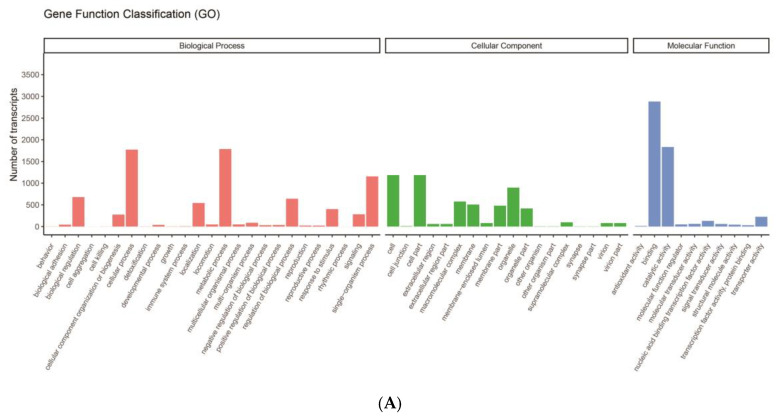

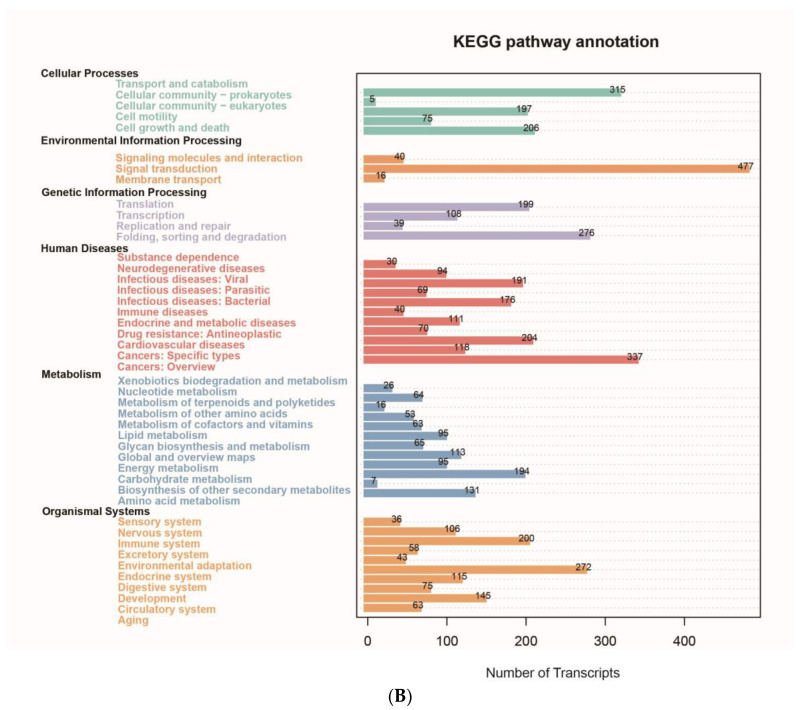
Functional annotation of transcripts of *N. denticulata sinensis*. (**A**) GO functional annotations. In total, 4136 transcripts were assigned to three categories: biological process, cellular component, and molecular function. *X*-axis represents different terms, and *Y*-axis represents number of transcripts annotated in corresponding term. (**B**) KEGG pathway functional annotations. In total, 5054 transcripts were annotated in KEGG database. *Y*-axis represents pathways under six primary classifications, and *X*-axis represents number of transcripts annotated in corresponding pathway.

The GO annotation revealed that binding (2882) and catalytic activity (1834) comprised large proportions in the molecular function category. Cell (1187), cell part (1187), and organelle (897) were the most represented terms in the cellular component category. In terms of the biological process category, most transcripts were involved in the metabolic process (1787), cellular process (1773), and single-organism process (1156) (Figure 2A).

KEGG is a comprehensive database that integrates genomic, chemical, and system function information. A KEGG pathway is a central database that provides information about molecular interaction, reaction, and related networks. Transcripts were divided into six classifications, including cellular processes, environmental information processing, genetic information processing, human diseases, metabolism, and organismal systems. Many genes were annotated in transport and catabolism (315), signal transduction (477), folding, sorting and degradation (276), carbohydrate metabolism (194), and endocrine system (272) (Figure 2B).

### 3.3. Alternative Splicing and Transcription Factor Analysis

The identified transcripts were mapped to 3661 genes. Skipped exon (261) is the most common type of alternative splicing event, and genes with exon skipping events account for 7.13% of the total annotated genes, followed by an alternative 5’ splice site (178, 4.86%), an alternative first exon (130, 3.55%), an alternative 3’ splice site (100, 2.73%), a retained intron (83, 2.27%), a mutually exclusive exon (38, 1.04%), and an alternative last exon (16, 0.44%).

Transcription factors are DNA-binding proteins that regulate gene transcription. A total of 39 transcription factor families containing 344 transcripts were identified. zf-C2H2 was the most annotated transcription factor family, including 108 transcripts. The ZBTB and HMG families comprise 53 and 38 transcripts, respectively.

### 3.4. Identification of LncRNAs

**Figure 3:**
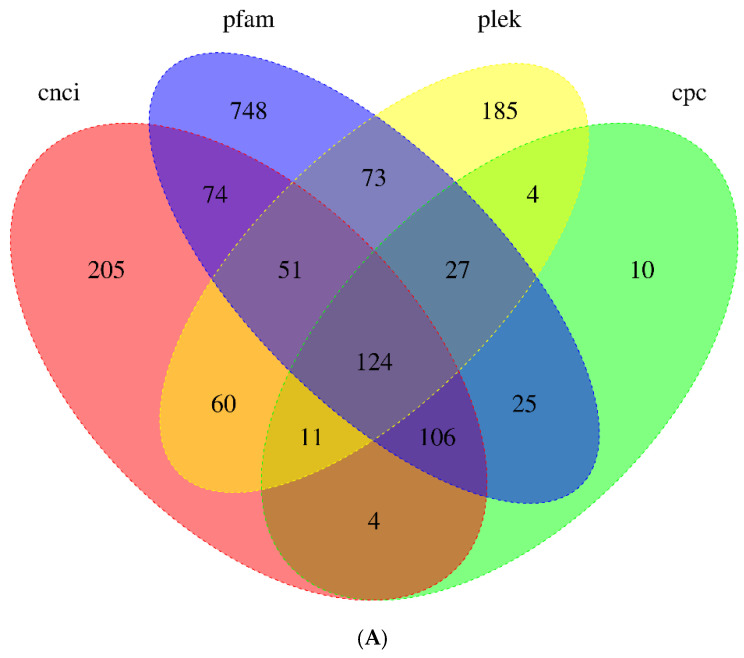

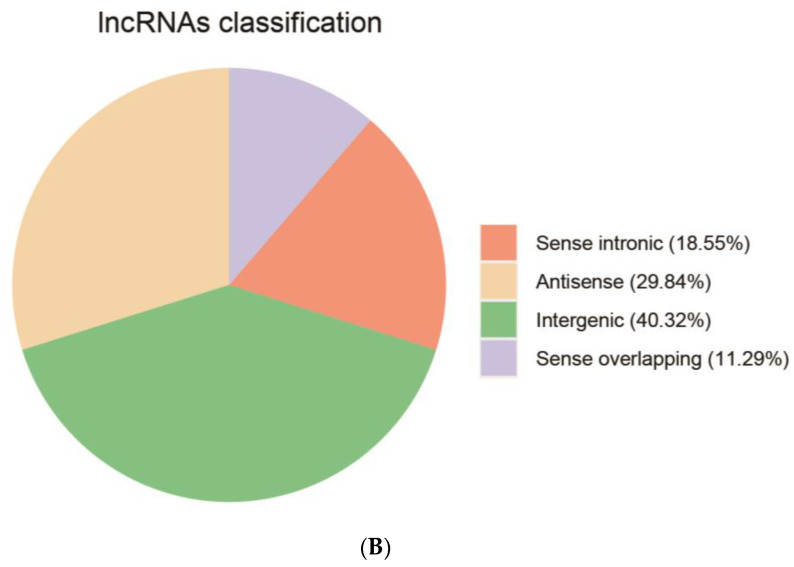
(**A**) The number of candidates LncRNAs identified using CNCI, PLEK, CPC, and Pfam. (**B**) The classification and proportion of LncRNAs based on genomic locations: sense intronic LncRNAs, antisense LncRNAs, intergenic LncRNAs, and sense overlapping LncRNAs.

LncRNAs are RNA molecules exceed 200 nt in length and are involved in nearly all biological processes and pathways [26]. In total, 635, 535, 311, and 1227 LncRNAs were predicted by the CNCI, PLEK, CPC, and Pfam databases, respectively (Figure 3A). A total of 124 transcripts were identified as LncRNAs using four prediction methods. The length of the longest LncRNA was 4237 nt, and the majority of the LncRNAs were about 1500 nt. LncRNAs were further categorized according to their genomic location: sense intronic (18.55%), sense overlapping (11.29%), antisense (29.84%), and intergenic (40.32%). Intergenic LncRNAs were the most prevalent type among them (Figure 3B).

### 3.5. Analysis of NdNHL-1

**Figure 4:**
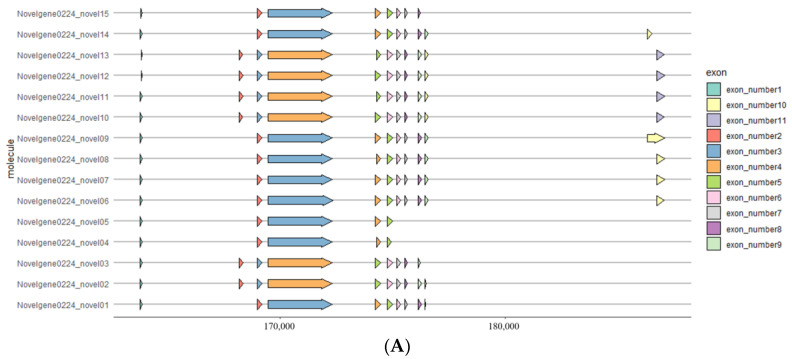

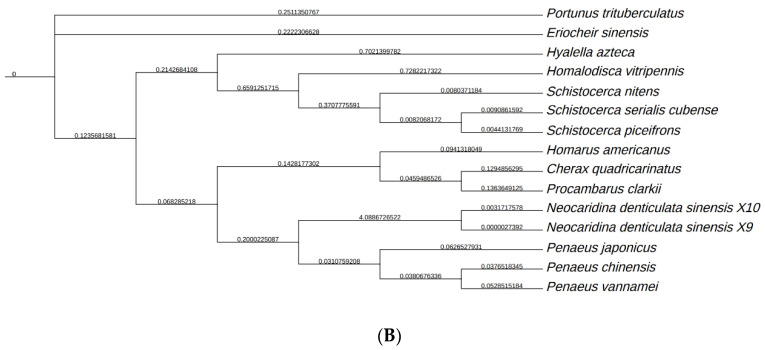
(**A**) Gene structure analysis of 15 *NdNHL-1* transcripts. Arrows with different colors represent different exon positions. (**B**) Phylogenetic analysis of NHL-1 from *N. denticulata sinensis* and other crustacean species using ML method. Values on branches represent evolutionary distance of NHL-1 in different species.

A predicted *NdNHL-1* was identified from *N. denticulata sinensis*. The longest transcript of *NdNHL-1* is 4995 bp and encodes a putative protein of 1665 amino acids. There are 15 transcripts of *NdNHL-1* based on alternative splicing annotation results; the longest transcript contains 11 exons, and the shortest contains only 8 exons (Figure 4A). The most abundant alternative splicing type in *NdNHL-1* is SE (skipping exon). Blast searches and amino acid sequence alignments revealed that NdNHL-1 is highly similar to NHL-1 in other arthropods. Novel 9 is most similar to NHL-1 X3 variable shear type III, and novel 10 is the standard NHL-1 gene. The constructed phylogenetic tree consists of three main clusters, and cluster 3 consists of four subclusters. Although there are many transcripts in *NdNHL-1*, it has more similarity to homologous proteins in shrimp (Figure 4B).

## 4. Discussion

Although next-generation sequencing technologies have been widely used in *N. denticulata sinensis*, it has inherent limitations such as GC bias, difficulties in mapping to complex parts of the genome, and phasing of variants [27]. Third-generation sequencing technologies, represented by the SMRT sequencing of PacBio and the nanopore sequencing of Oxford Nanopore Technologies, permit the production of exceptionally long reads and the identification of isoform features [28]. SMRT sequencing has been applied in transcriptome studies of aquatic organisms, such as *L. vannamei* [29], *C. sapidus* [30], and *Platypharodon extremus* [31]. The present study described a full-length cDNA library from multiple tissues of *N. denticulata sinensis*. A total of 63.96 Gb subreads were obtained, resulting in 193,468 CCSs and 5831 transcripts. A total of 90.5% of the transcripts were novel, highlighting the potential of long-read sequencing to identify new isoforms. Regarding the Illumina sequencing results, the N50 value of the unigenes ranged from 1513 bp to 2079 bp, and the mean length of the unigenes was about 1000 bp [32,33]. Based on the SMRT sequencing results, the N50 value and mean length of transcripts were 3697 bp and 3167 bp, respectively, which are significantly longer than the second-generation sequencing data [32].

Transcription factors recognize specific DNA sequences to guide the expression of the genome, and they are vital for the normal development of an organism. Mutations in transcription factors often cause many human diseases [34]. In this study, 29 transcription factor families were identified, and the top 5 transcription factor families were zf-C2H2, ZBTB, HMG, Homeobox, and ARID. There are diverse types of zinc finger proteins (ZNFs), and the zf-C2H2 family is the largest one. As it is involved in diverse and important biological functions and is exposed to strong evolutional pressure, zf-C2H2 is present throughout organisms from yeasts to humans [35]. In *Larimichthys crocea*, zf-C2H2 may play an important role in response to hypoxia and acidification stress [36]. Studies have also shown that a high expression of the zf-C2H2 gene may enhance the tolerance of *Ruditapes philippinarum* to *Vibrio anguillarum* infection [37]. Additionally, ZBTB proteins are important members of the ZNF family and are better characterized in mammals, such as lymphoid development, myeloid development, and diseases [38,39,40]. Considering the multi-function and higher annotation ratio, zf-C2H2 was expected to play a key role in the environmental adaptability of *N. denticulata sinensis*.

A significant portion of the genome consists of non-coding regulatory elements. Increasing evidence shows that many non-coding RNAs play significant regulatory roles in complex organisms [41]. Indeed, LncRNAs has become a growing focus of genomic studies in recent years. Many studies have found that LncRNA participates in a variety of biological processes, including protein complex assembly, chromatin organization, and transcriptional and post-transcriptional gene regulation [42,43]. In the present study, 124 LncRNAs were identified from *N. denticulata sinensis*, and most of them were classified as long intergenic non-coding RNAs. It is thought that long intergenic non-coding RNAs are the largest subclass of non-coding RNAs in the human genome [44]. Moreover, the higher proportion of lincRNAs is consistent with the results in studies of other insects [45,46].

Alternative splicing generates multiple transcripts from a single gene, enriching the diversity of proteins and phenotypic traits; it can facilitate adaptation as well as contribute to species divergence [47]. In invertebrates, alternative splicing has proven to be involved in various processes, for instance, neuronal development, immune specificity, and sex determination [48,49,50,51]. A total of 1236 alternative splicing events from 806 genes were predicted in this study, occupying 22% of the total genes identified. This is close to the ratio reported in *Drosophila* and *Apis mellifera* [52,53], indicating that alternative splicing is a common step in post-transcriptional gene regulation.

Multiple transcripts were assigned to environmental information processing and organismal systems. The endocrine system and immune system were two mainly secondary items of organismal systems. The primary pathways involved in these items include the thyroid hormone signaling pathway (ko04919), insulin signaling pathway (ko04910), glucagon signaling pathway (ko04922), oxytocin signaling pathway (ko04921), chemokine signaling pathway (ko04062), NOD-like receptor signaling pathway (ko04621), Toll and IMD signaling pathway (ko04624), and antigen processing and presentation (ko04612). It is generally accepted that the crustacean neuroendocrine–immune system plays a fundamental role in maintaining homeostasis and environmental adaptability [54]. In *L. vannamei*, 5-HT and acetylcholine could regulate the immune defense of hemocytes under ammonia-N stress [55]. Similarly, B-type allatostatin was proven to induce an innate immune response in the mud crab [56]. The pathways identified here lay a theoretical foundation for the research of crustacean immunity and environmental adaptability.

NHL is a conserved structural motif found in a large family of growth regulators. Many NHL-containing proteins have additional domains, such as a RING finger, a B-box zinc finger, or a coiled-coil motif. A structural modeling analysis suggests that the NHL domain may be involved in protein–protein interaction [57]. The NHL domain is a six-bladed beta-propeller, with the blades arranged radially around a central axis, and each blade consists of a highly twisted four-stranded antiparallel beta-sheet [57]. The innermost strand of each blade is labeled a″, and the outermost strand is labeled d″. As in other beta propellers, the sequence repeats are offset with respect to the blades of the propeller, so that any given 40-residue NHL repeat spans the b′–d′ strands of one propeller blade and the a″ strand of the following blade [58]. This offset ensures the circularization of the propeller because the last strand of the final sequence repeat acts as an innermost strand of the blade that harbors strands b′–d′ from the first sequence repeat. Compared with other members in the RING finger protein family, NHL-1 is a less commonly discussed one [12]. In *Caenorhabditis elegans*, NHL-1 acts in chemosensory neurons to promote stress resistance in distal tissues through the activation of the transcription factor DAF-16 [59]. The *NHL-1* we found contained repeats of the entire NHL sequence and was identified for the first time in *N. denticulata sinensis*. Whether *NdNHL*-1 is involved in stress resistance remains unclear, but the Iso-seq results provide a better opportunity for understanding its gene structure and lays the groundwork for future experimental research.

## 5. Conclusions

The present study assembled a comprehensive full-length transcriptome of *N. denticulata sinensis*, encompassing 5831 transcripts, a majority (90.5%) of which revealed new isoforms of known genes. The key findings also included the discovery of 1236 alternative splicing events, 344 transcription factors, and 124 LncRNAs, highlighting the genomic complexity and regulatory diversity in this species. Moreover, *NdNHL-1* was characterized with 15 transcripts, and a phylogenetic analysis of NdNHL-1 contributes to our understanding of gene evolution within crustaceans. These findings enrich the genetic resources of *N. denticulata sinensis* from the isoform level, which will promote the discovery and functional studies of novel genes, as well as benefit potential applications in crustacean biology and aquaculture.

## Figures and Tables

**Table 1:** Characteristics of Iso-seq sequences of *N. denticulata sinensis*.

Type	Number	Minimum Length	Maximum Length	Mean Length	N50
Polymerase read	548,606			119,673	192,219
Subread	23,201,449			2757	3237
CCS	193,468	74	16,527	3243	3640
FLNC read	154,402	52	14,041	3145	3559
Consensus sequence	13,082	83	9788	3262	3749
Isoform sequence	5831			3166.76	3697

**Table 2:** Species classification of transcripts based on NR annotation (species were ranked according to homology of *N. denticulate sinensis* to specific species, “Transcripts number” represents number of transcripts that showed homologous hits to corresponding species, and “%” indicates proportion of homologous transcripts to all transcripts).

Rank	Species	Transcript Number	%
1	*Hyalella azteca*	2224	42.05
2	*Zootermopsis nevadensis*	359	6.79
3	*Limulus polyphemus*	164	3.1
4	*Litopenaeus vannamei*	124	2.34
5	*Daphnia magna*	112	2.12
6	*Penaeus monodon*	88	1.66
7	others	2200	41.6

## Data Availability

The raw sequence data reported in this study were deposited in the Genome Sequence Archive in the National Genomics Data Center [60], the China National Center for Bioinformation/Beijing Institute of Genomics, the Chinese Academy of Sciences under accession code CRA016316 (shared URL: https://ngdc.cncb.ac.cn/gsa/s/2Hj152U8 (accessed on 15 April 2024)), or they can be accessed via the National Center for Biotechnology Information’s (NCBI) Sequence Read Archive (SRA) database under accession number SRP508239.

## References

[1] Mykles D.L., Hui J.H. (2015). *Neocaridina denticulata*: A Decapod Crustacean Model for Functional Genomics. Integr. Comp. Biol..

[2] Tang C.H., Chen W.Y., Wu C.C., Lu E., Shih W.Y., Chen J.W., Tsai J.W. (2020). Ecosystem metabolism regulates seasonal bioaccumulation of metals in atyid shrimp (*Neocaridina denticulata*) in a tropical brackish wetland. Aquat. Toxicol..

[3] Liang M., Ma L., Li X., Feng D., Zhang J., Sun Y. (2022). Identification and characterization of two types of triacylglycerol lipase genes from *Neocaridina denticulata sinensis*. Fish Shellfish Immun..

[4] Gallardo-Escarate C., Valenzuela-Munoz V., Nunez-Acuna G., Valenzuela-Miranda D., Goncalves A.T., Escobar-Sepulveda H., Liachko I., Nelson B., Roberts S., Warren W. (2021). Chromosome-scale genome assembly of the sea louse *Caligus rogercresseyi* by SMRT sequencing and Hi-C analysis. Sci. Data.

[5] Chen J., Yu Y., Kang K., Zhang D. (2020). SMRT sequencing of the full-length transcriptome of the white-backed planthopper *Sogatella furcifera*. PeerJ.

[6] Zhang Y., Lou F., Chen J., Han Z., Yang T., Gao T., Song N. (2022). Single-molecule Real-time (SMRT) Sequencing Facilitates Transcriptome Research and Genome Annotation of the Fish *Sillago sinica*. Mar. Biotechnol..

[7] Zhao L., Zhang H., Kohnen M.V., Prasad K., Gu L., Reddy A.S.N. (2019). Analysis of Transcriptome and Epitranscriptome in Plants Using PacBio Iso-Seq and Nanopore-Based Direct RNA Sequencing. Front. Genet..

[8] Pootakham W., Uengwetwanit T., Sonthirod C., Sittikankaew K., Karoonuthaisiri N. (2020). A novel full-length transcriptome resource for black tiger shrimp (*Penaeus monodon*) developed using isoform sequencing (Iso-Seq). Front. Mar. Sci..

[9] Zhang Y., Ni M.Q., Bai Y.H., Shi Q., Zheng J.B., Cui Z.X. (2022). Full-length transcriptome analysis provides new insights into the diversity of immune-related genes in *Portunus trituberculatus*. Front. Immunol..

[10] Wan H., Jia X., Zou P., Zhang Z., Wang Y. (2019). The Single-molecule long-read sequencing of *Scylla paramamosain*. Sci. Rep..

[11] Zhao J., He Z., Chen X., Huang Y., Xie J., Qin X., Ni Z., Sun C. (2021). Growth trait gene analysis of kuruma shrimp (*Marsupenaeus japonicus*) by transcriptome study. Comp. Biochem. Physiol. Part D Genom. Proteom..

[12] Cai C.M., Tang Y.D., Zhai J.B., Zheng C.F. (2022). The RING finger protein family in health and disease. Signal Transduct. Target. Ther..

[13] Salmela L., Rivals E. (2014). LoRDEC: Accurate and efficient long read error correction. Bioinformatics.

[14] Wu T.D., Watanabe C.K. (2005). GMAP: A genomic mapping and alignment program for mRNA and EST sequences. Bioinformatics.

[15] Young M.D., Wakefield M.J., Smyth G.K., Oshlack A. (2010). Gene ontology analysis for RNA-seq: Accounting for selection bias. Genome Biol..

[16] Mao X.Z., Cai T., Olyarchuk J.G., Wei L.P. (2005). Automated genome annotation and pathway identification using the KEGG Orthology (KO) as a controlled vocabulary. Bioinformatics.

[17] Alamancos G.P., Pagès A., Trincado J.L., Bellora N., Eyras E. (2015). Leveraging transcript quantification for fast computation of alternative splicing profiles. RNA.

[18] Zhang H.M., Liu T., Liu C.J., Song S.Y., Zhang X.T., Liu W., Jia H.B., Xue Y., Guo A.Y. (2015). AnimalTFDB 2.0: A resource for expression, prediction and functional study of animal transcription factors. Nucleic Acids Res..

[19] Sun L., Luo H.T., Bu D.C., Zhao G.G., Yu K.T., Zhang C.H., Liu Y.N., Chen R.S., Zhao Y. (2013). Utilizing sequence intrinsic composition to classify protein-coding and long non-coding transcripts. Nucleic Acids Res..

[20] Finn R.D., Coggill P., Eberhardt R.Y., Eddy S.R., Mistry J., Mitchell A.L., Potter S.C., Punta M., Qureshi M., Sangrador-Vegas A. (2016). The Pfam protein families database: Towards a more sustainable future. Nucleic Acids Res..

[21] Li A.M., Zhang J.Y., Zhou Z.Y. (2014). PLEK: A tool for predicting long non-coding RNAs and messenger RNAs based on an improved k-mer scheme. BMC Bioinform..

[22] Kong L., Zhang Y., Ye Z.Q., Liu X.Q., Zhao S.Q., Wei L., Gao G. (2007). CPC: Assess the protein-coding potential of transcripts using sequence features and support vector machine. Nucleic Acids Res..

[23] Wilkins D. (2023). gggenes: Draw Gene Arrow Maps in ‘ggplot2’. https://wilkox.org/gggenes/.

[24] Rozewicki J., Li S., Amada K.M., Standley D.M., Katoh K. (2019). MAFFT-DASH: Integrated protein sequence and structural alignment. Nucleic Acids Res..

[25] Nguyen L.T., Schmidt H.A., von Haeseler A., Minh B.Q. (2015). IQ-TREE: A fast and effective stochastic algorithm for estimating maximum-likelihood phylogenies. Mol. Biol. Evol..

[26] Li Y., Yang X.J., Kang X.N., Liu S.L. (2019). The regulatory roles of long noncoding RNAs in the biological behavior of pancreatic cancer. Saudi J. Gastroenterol..

[27] Ardui S., Ameur A., Vermeesch J.R., Hestand M.S. (2018). Single molecule real-time (SMRT) sequencing comes of age: Applications and utilities for medical diagnostics. Nucleic Acids Res..

[28] Byrne A., Cole C., Volden R., Vollmers C. (2019). Realizing the potential of full-length transcriptome sequencing. Philos. Trans. R. Soc. B.

[29] Zeng D., Chen X., Peng J., Yang C., Peng M., Zhu W., Xie D., He P., Wei P., Lin Y. (2018). Single-molecule long-read sequencing facilitates shrimp transcriptome research. Sci. Rep..

[30] Gao B.Q., Lv J.J., Meng X.L., Li J.T., Li Y.K., Liu P., Li J. (2022). Full-length transcriptome construction of the blue crab *Callinectes sapidus*. Front. Mar. Sci..

[31] Wu X., Gong Q., Chen Y., Liu Y., Song M., Li F., Li P., Lai J. (2022). Full-length transcriptome and analysis of bmp-related genes in *Platypharodon extremus*. Heliyon.

[32] Xing K.F., Liu Y.J., Yan C.C., Zhou Y.Z., Sun Y.Y., Su N.K., Yang F.S., Xie S., Zhang J.Q. (2021). Transcriptome analysis of *Neocaridina denticulate sinensis* under copper exposure. Gene.

[33] Liu Y.J., Xing K.F., Yan C.C., Zhou Y.Z., Xu X.M., Sun Y.Y., Zhang J.Q. (2022). Transcriptome analysis of *Neocaridina denticulate sinensis* challenged by *Vibrio parahemolyticus*. Fish Shellfish Immun..

[34] Lambert S.A., Jolma A., Campitelli L.F., Das P.K., Yin Y.M., Albu M., Chen X.T., Taipale J., Hughes T.R., Weirauch M.T. (2018). The Human Transcription Factors. Cell.

[35] Mackeh R., Marr A.K., Fadda A., Kino T. (2018). C2H2-Type Zinc Finger Proteins: Evolutionarily Old and New Partners of the Nuclear Hormone Receptors. Nucl. Recept. Signal..

[36] Wang Y., Chen R., Wang Q., Yue Y., Gao Q., Wang C., Zheng H., Peng S. (2022). Transcriptomic analysis of large yellow croaker (*Larimichthys crocea*) during early development under hypoxia and acidification stress. Vet. Sci..

[37] Yin Z., Nie H., Jiang K., Yan X. (2022). Molecular Mechanisms Underlying *Vibrio* Tolerance in *Ruditapes philippinarum* Revealed by Comparative Transcriptome Profiling. Front. Immunol..

[38] Lee S.U., Maeda T. (2012). POK/ZBTB proteins: An emerging family of proteins that regulate lymphoid development and function. Immunol. Rev..

[39] Zhang Z.S., Wu L.L., Li J., Chen J.Y., Yu Q., Yao H., Xu Y.P., Liu L. (2022). Identification of ZBTB9 as a potential therapeutic target against dysregulation of tumor cells proliferation and a novel biomarker in Liver Hepatocellular Carcinoma. J. Transl. Med..

[40] Masuda T., Wang X., Maeda M., Canver M.C., Sher F., Funnell A.P.W., Fisher C., Suciu M., Martyn G.E., Norton L.J. (2016). Transcription factors LRF and BCL11A independently repress expression of fetal hemoglobin. Science.

[41] Wang K.C., Chang H.Y. (2011). Molecular mechanisms of long noncoding RNAs. Mol. Cell.

[42] Morlando M., Ballarino M., Fatica A. (2015). Long Non-Coding RNAs: New Players in Hematopoiesis and Leukemia. Front. Med..

[43] Sun Q., Hao Q., Prasanth K.V. (2018). Nuclear Long Noncoding RNAs: Key Regulators of Gene Expression. Trends Genet..

[44] Cabili M.N., Trapnell C., Goff L., Koziol M., Tazon-Vega B., Regev A., Rinn J.L. (2011). Integrative annotation of human large intergenic noncoding RNAs reveals global properties and specific subclasses. Genes Dev..

[45] Azlan A., Obeidat S.M., Das K.T., Yunus M.A., Azzam G. (2021). Genome-wide identification of *Aedes albopictus* long noncoding RNAs and their association with dengue and Zika virus infection. PLoS Neglected Trop. Dis..

[46] Wu Y., Cheng T., Liu C., Liu D., Zhang Q., Long R., Zhao P., Xia Q. (2016). Systematic Identification and Characterization of Long Non-Coding RNAs in the Silkworm, *Bombyx mori*. PLoS ONE.

[47] Wright C.J., Smith C.W.J., Jiggins C.D. (2022). Alternative splicing as a source of phenotypic diversity. Nat. Rev. Genet..

[48] Mohr C., Hartmann B. (2014). Alternative splicing in *Drosophila* neuronal development. J. Neurogenet..

[49] Riddell C.E., Lobaton Garces J.D., Adams S., Barribeau S.M., Twell D., Mallon E.B. (2014). Differential gene expression and alternative splicing in insect immune specificity. BMC Genom..

[50] Salz H.K. (2011). Sex determination in insects: A binary decision based on alternative splicing. Curr. Opin. Genet. Dev..

[51] Xu Y., Yang Y., Zheng J., Cui Z. (2022). Alternative splicing derived invertebrate variable lymphocyte receptor displays diversity and specificity in immune system of crab *Eriocheir sinensis*. Front. Immunol..

[52] Gibilisco L., Zhou Q., Mahajan S., Bachtrog D. (2016). Alternative splicing within and between *Drosophila* species, sexes, tissues, and developmental stages. PLoS Genet..

[53] Zheng S.Y., Pan L.X., Cheng F.P., Jin M.J., Wang Z.L. (2023). A global survey of the full-length transcriptome of *Apis mellifera* by single-molecule long-read sequencing. Int. J. Mol. Sci..

[54] Tong R.X., Pan L.Q., Zhang X., Li Y.F. (2022). Neuroendocrine-immune regulation mechanism in crustaceans: A review. Rev. Aquac..

[55] Zhang X., Pan L.Q., Tong R.X., Li Y.F., Tian Y.M., Li D.Y., Si L.J. (2021). PacBio full length transcript sequencing and Illumina transcriptome insight into immune defense mechanism of *Litopenaeus vannamei* under ammonia-N stress. Aquaculture.

[56] Xu Z., Wei Y., Wang G., Ye H. (2021). B-type allatostatin regulates immune response of hemocytes in mud crab *Scylla paramamosain*. Dev. Comp. Immunol..

[57] Edwards T.A., Wilkinson B.D., Wharton R.P., Aggarwal A.K. (2003). Model of the brain tumor-Pumilio translation repressor complex. Genes Dev..

[58] Slack F.J., Ruvkun G. (1998). A novel repeat domain that is often associated with RING finger and B-box motifs. Trends Biochem. Sci..

[59] Volovik Y., Moll L., Marques F.C., Maman M., Bejerano-Sagie M., Cohen E. (2014). Differential regulation of the heat shock factor 1 and DAF-16 by neuronal nhl-1 in the nematode *C. elegans*. Cell Rep..

[60] Xu X.J., Hao L.L., Zhu J.W., Zhou Q., Song F.H., Chen T.T., Zhang S.S., Dong L.L., Lan L., Wang Y.Q. (2018). Database Resources of the BIG Data Center in 2018. Nucleic Acids Res..

